# Association of *Helicobacter pylori* Infection with the Gastric Microbiota: A Scoping Review from a Molecular Diagnostic Perspective

**DOI:** 10.3390/microorganisms14102137

**Published:** 2026-09-23

**Authors:** Christel Paulina Quiñonez Álvarez, Lennys Berutti-Suárez, Santiago Xavier Mafla Andrade

**Affiliations:** 1 Maestría en Microbiología con Mención en Microbiología Aplicada, Posgrado, Universidad Politécnica Estatal del Carchi (UPEC), Tulcán 040150, Ecuador; christel.quinonez@upec.edu.ec (C.P.Q.Á.); sxmafla@pucesi.edu.ec (S.X.M.A.); 2 Carrera de Laboratorio Clínico, Facultad de Ciencias de la Salud y Ciencias de la Educación, Universidad Politécnica Estatal del Carchi (UPEC), Tulcán 040150, Ecuador; 3 Escuela de Ciencias Agropecuarias y Ambientales, Pontificia Universidad Católica del Ecuador Sede Ibarra (PUCE-I), Ibarra 100112, Ecuador

**Keywords:** qPCR, 16S rRNA sequencing, shotgun metagenomics, dysbiosis, gastric cancer

## Abstract

*Helicobacter pylori* infection remains globally one of the main causes of chronic gastritis, gastroduodenal ulcer disease, and gastric adenocarcinoma. Although the stomach has been considered an almost sterile environment, molecular techniques have revealed a complex and dynamic gastric microbiome whose composition may vary with *H. pylori* infection status. This review presents current knowledge on the impact of *H. pylori* on the composition, diversity, and functional implications of the gastric microbiome, focusing on studies applying molecular diagnostic techniques such as conventional PCR, quantitative real-time PCR (qPCR), 16S rRNA gene sequencing, and shotgun metagenomics. A systematic literature search was conducted between January and March 2025, including all eligible studies published from 2006 to 2025 following the PRISMA methodology for exploratory reviews. Thirty studies met the inclusion criteria and were integrated into the qualitative synthesis, which included primary studies, secondary sources, and methodological studies. The evidence suggests that *H. pylori* infection is generally associated with decreased alpha diversity and increased relative abundance of Proteobacteria (especially Helicobacter), along with decreased abundances of *Firmicutes*, *Actinobacteria*, and *Bacteroidetes*. Several studies have reported associations between virulence factors (e.g., CagA and VacA) and alterations in the gastric microenvironment, reflecting microbial and host-related changes relevant to chronic inflammation and gastric carcinogenesis. From a diagnostic perspective, qPCR and sequencing-based methods enable detection of *H. pylori*, evaluation of virulence- and resistance-associated targets, and broader taxonomic and functional analyses. However, significant methodological heterogeneity, particularly concerning sample types, DNA extraction, 16S hypervariable regions, and bioinformatics platforms, limits comparability between studies. These findings underscore the need for standardized molecular methods to improve understanding of interactions between *H. pylori* and the microbiome, as well as their clinical implications.

## 1. Introduction

Recent molecular techniques have revealed a diverse and dynamic gastric microbiota. It contains a variety of bacterial groups that contribute to essential physiological, metabolic, and immunological processes that support gastrointestinal homeostasis [1,2,3]. These findings challenge the traditional understanding of the gastric environment and open new lines of research focusing on microbe–host interactions in digestive diseases.

*Helicobacter pylori* has significant clinical importance due to its worldwide prevalence and its association with pathologies such as chronic gastritis, gastroduodenal ulcers, gastric mucosa-associated lymphoid tissue (MALT) lymphoma, and gastric cancer [4,5]. Currently, approximately 50% of the world’s population is infected with *H. pylori*, with a higher prevalence in developing countries where sanitary and socioeconomic conditions favor transmission [4].

The persistence of *H. pylori* in the gastric environment is supported by adaptive mechanisms, including urease production, adhesion to epithelial cells, and the expression of virulence factors that modulate the host immune response and enable survival under highly acidic conditions [6,7,8,9,10]. These characteristics also facilitate long-term colonization of the gastric mucosa and contribute to chronic inflammatory processes.

Recent research has shown that *H. pylori* infection is associated with alterations in the composition and diversity of the gastric microbiota [2,11,12]. Sequencing analyses have revealed reduced alpha diversity and community changes, characterized by dominance of *H. pylori* and shifts in other taxa, although the direction and magnitude of these changes vary across populations and study designs [9,13,14,15,16]. Other studies have reported reduced levels of *Firmicutes*, *Actinobacteria*, and *Bacteroidetes* in *H. pylori*-positive individuals, which is consistent with dysbiosis associated with chronic inflammation [12,15,16]. These variations may be influenced by factors such as age, diet, proton pump inhibitor use, previous antibiotic exposure, and the host’s immune status [17].

Proteins such as CagA and VacA have been implicated in epithelial damage, alteration in signaling pathways, and immunomodulation that contribute to inflammation associated with gastric carcinogenesis. They are considered bacterial virulence factors, essential for shaping the gastric microenvironment [13,14,18,19]. These changes in the host and mucosa can create ecological conditions that favor alterations in the structure and function of the gastric microbial ecosystem.

Recent advances in molecular diagnostics have significantly improved the study of *H. pylori* and the gastric microbiota. Molecular techniques, such as PCR, are widely used to detect *H. pylori* and to identify clinically relevant targets, including virulence genes and mutations associated with antibiotic resistance [4,20]. Furthermore, 16S rRNA gene sequencing and shotgun metagenomics enable comprehensive characterization of microbial communities, including detection of uncultivable microorganisms and analysis of functional profiles associated with disease development [7,9,21,22].

On the other hand, methodological variability remains a limitation for comparability among the numerous reported studies. The main sources of heterogeneity include differences in sample type and anatomical site, DNA extraction protocols, and hypervariable regions targeted for 16S rRNA amplification, sequencing depth, and bioinformatics workflows for taxonomic assignment [11,23,24]. The low biomass of gastric samples further increases the risk of contamination bias if extraction blanks and appropriate controls are not implemented [24]. These challenges complicate data synthesis and partly explain the inconsistent results in the literature.

In this regard, this structured literature review enables the identification of research trends, the most common molecular methods, and the main findings regarding the association between *H. pylori* and the gastric microbiota. Furthermore, it summarizes the current evidence on how *H. pylori* infection status is associated with the composition, diversity, and functional potential of the gastric microbiota from the perspective of molecular diagnostics, emphasizing the roles of PCR, quantitative PCR (qPCR), 16S rRNA gene sequencing, and shotgun metagenomics, as well as the main methodological strengths and limitations reported in the reviewed studies.

Unlike previous reviews that focused primarily on the biological and clinical consequences of gastric dysbiosis associated with *Helicobacter pylori* [2,3,4,21,22,25], this review adopts a conceptual framework centered on molecular diagnosis. It systematizes and compares the analytical performance, applications, and limitations of the main tools employed (conventional PCR, qPCR, 16S rRNA gene sequencing, and shotgun metagenomics). Additionally, it incorporates an explicit classification of the type of evidence (primary study, secondary source, or methodological study) for each included source, in order to more precisely define the origin and level of certainty of the reported findings, an aspect that has not been systematically addressed in previous reviews on this topic.

## 2. Materials and Methods

### 2.1. Study Design

This scoping review was conducted and reported in accordance with the PRISMA Extension for Scoping Reviews (PRISMA-ScR) guidelines [26]. The objective was to map the available evidence, identify research gaps, and describe the methodological profile of studies that used molecular diagnostic methods to examine associations between *Helicobacter pylori* infection and the gastric microbiota. Aggregate effect size was not estimated. Although this review was not prospectively registered, the inclusion criteria, search strategy, and data extraction variables were predefined and documented before study selection began.

### 2.2. Research Question (PEO Framework)

The scope of the study was defined, and the bibliographic research was guided by the Population–Exposure–Outcome (PEO) framework, adapted for scoping reviews in microbiology.

- Population: Studies including individuals with or without *Helicobacter pylori* infection in which the gastric microbiome was evaluated.
- Exposure: Presence of *Helicobacter pylori* infection, assessed using molecular diagnostic tools such as PCR, qPCR, 16S rRNA gene sequencing, shotgun metagenomics, or combinations thereof.
- Outcomes: Alterations in the composition, alpha and beta diversity, and functional profile of the gastric microbiome; findings concerning bacterial virulence factors (CagA, VacA, OipA); and methodological characteristics of the molecular techniques utilized.

This framework informed the formulation of the following research question:

What evidence have studies employing molecular diagnostic tools reported regarding associations between *Helicobacter pylori* infection and the composition and function of the gastric microbiome?

### 2.3. Bibliographic Search Strategy

An exhaustive bibliographic search was conducted in four electronic databases: PubMed, Scopus, Web of Science, and SciELO between January and March 2025. The search strategy combined controlled vocabulary (MeSH terms in PubMed; subject headings where available) with free-text keywords in English and Spanish, covering *H. pylori*, gastric microbiota, dysbiosis, and molecular diagnostic methods.

Key search terms included “*Helicobacter pylori*”, “gastric microbiota”, “gastric microbiome”, “gastric dysbiosis”, “molecular diagnosis”, “PCR”, “qPCR”, “16S rRNA sequencing”, “next-generation sequencing”, “shotgun metagenomics”, and “virulence factors”. Boolean operators (AND/OR) were used to combine terms. The full search strings per database are shown in Table 1.

Publications from 2020 to 2025 were prioritized to capture recent advances in sequencing-based microbiota research. Studies from 2006 to 2019 were also retained if they provided foundational methodological or pathophysiological evidence essential to the context. No language restrictions beyond English and Spanish were applied. Reference lists of eligible full-text articles were manually screened to identify additional relevant records not captured by the electronic search.

### 2.4. Eligibility Criteria

Eligibility criteria were defined according to the PEO framework. Table 2 below shows only a few selected examples; the full, unedited list of included studies is presented in Supplementary Table S1. Additionally, to transparently distinguish the level of evidence throughout this review, each included source was classified as: P (Primary empirical study, original data collection), S (Secondary source, narrative review, systematic review, meta-analysis, or consensus document), or M (Methodological study). This classification is presented in a dedicated column in Table 2. Of the 30 included sources, 17 were classified as primary empirical studies, 11 as secondary synthesis sources, and 2 as methodological contributions. This labeling allows the reader to identify, at any point in the manuscript, whether a reported finding comes from first-hand data or from a synthesis of previous studies, directly addressing the risk of conceptual duplication that can arise when primary findings and their subsequent syntheses are cited together.

#### 2.4.1. Inclusion Criteria

Original studies (observational, cross-sectional, Case–control, cohort), systematic reviews, and meta-analyses on the interaction between *Helicobacter pylori* and the gastric microbiota.

Studies using at least one molecular diagnostic tool (conventional PCR, quantitative PCR (qPCR), 16S rRNA gene sequencing, or shotgun metagenomics).

Studies reporting data on bacterial diversity, taxonomic composition, virulence factors (CagA, VacA, OipA), and/or the impact of eradication therapy on the gastric microbiota.

Publications in English or Spanish, with full text available.

No restrictions on geographical origin or clinical setting.

#### 2.4.2. Exclusion Criteria

Duplicate records.

Studies that do not directly assess the interaction between *H. pylori* and the gastric microbiota (e.g., those limited to the intestinal microbiota without a gastric component, or those focused exclusively on gastric conditions unrelated to *H. pylori*).

Studies that do not directly assess the interaction between *H. pylori* and the gastric microbiota (e.g., studies limited to the intestinal microbiota without assessing *H. pylori* infection/eradication status and without linking findings to *H. pylori*-related gastric outcomes). Fecal-only studies evaluating microbiome perturbations in the context of *H. pylori* eradication, or gut microbiota associated with *H. pylori*-related gastric lesions, were retained and interpreted as responses across compartments rather than as direct measures of the gastric mucosal community.

Publications for which the full text was not available after contacting the corresponding author.

Conference abstracts, editorials, letters, and opinion pieces that did not contain original data.

#### 2.4.3. Inclusion of Secondary Sources and Methodological Studies

In line with the exploratory and comprehensive nature of scoping reviews [26], this review included not only primary empirical studies (observational, cross-sectional, Case–control, cohort studies) but also secondary sources, such as narrative reviews, systematic reviews, meta-analyses, and international consensus documents. The conditions were that: (a) they directly related to the interaction between *Helicobacter pylori* and the gastric microbiota; (b) they described or evaluated molecular diagnostic tools; and (c) they provided the necessary contextual, methodological, or pathophysiological basis for interpreting primary evidence. Examples include the reviews [2,3,25], the international clinical consensus [4], and the methodological contributions [1,21]. These sources were classified as secondary evidence in the data extraction form (Supplementary Table S1) and treated separately from primary empirical studies in the qualitative synthesis. Their inclusion aligns with the established methodology of scoping reviews, which prioritizes breadth of evidence mapping over restrictions to primary research designs [26].

Furthermore, two methodological studies [6,24] were retained despite not being specific to *H. pylori* or the gastric microbiota. Their inclusion was justified because they established an analytical framework for characterizing low-abundance microbiota in clinical samples via whole-genome sequencing, a challenge directly relevant to gastric studies [24]; (b) they introduced barcoded pyrosequencing as a comparative approach for gastrointestinal microbiota analysis, becoming a technical benchmark for later 16S rRNA-based gastric microbiota studies [6]. Both studies contributed only to the methodological characterization component of the review and were clearly labeled as such in Supplementary Table S1.

### 2.5. Study Selection Process

Two reviewers (C.P.Q.Á. and L.B.-S.) independently selected studies in three steps, following PRISMA-ScR guidelines [26]. They discussed any disagreements, and if they could not agree, a third reviewer (S.X.M.A.) made the final decision.

Stage 1: Title screening. After removing 32 duplicates, 148 unique records were screened by title. Studies clearly outside the review’s scope, such as those on extragastric sites, veterinary-only studies, or non-molecular methods, were excluded.

Stage 2: Abstract screening. The abstracts of the remaining records were checked against the inclusion criteria. Records that did not meet at least one criterion were excluded at this stage (*n* = 84).

Stage 3: Full-text evaluation. Sixty-four full articles were retrieved and reviewed independently. Articles were excluded for reasons listed in Supplementary Table S1: not enough methodological detail to answer the research question (*n* = 12), not reporting direct interactions between *Helicobacter pylori* and the gastric microbiota (*n* = 9), not enough detail on molecular diagnostic methods (*n* = 7), or the full text was still unavailable after contacting the author (*n* = 6).

In the end, 30 sources met all the eligibility criteria and were included in the qualitative synthesis. Figure 1 shows the full selection process.

When needed, we standardized molecular techniques, sample types, and groups of microorganisms across the included sources. We documented these choices during data extraction to keep the process transparent and reproducible. We did not formally assess methodological quality or risk of bias, as this review aims to describe the scope and features of the available evidence, not to estimate intervention effects or compare outcomes across studies [26].

### 2.6. Data Charting Process

Two reviewers (C.P.Q.Á. and L.B.-S.) independently extracted data using a standardized data mapping form that was developed and pilot-tested on five randomly selected studies. The form underwent iterative refinement to ensure comprehensive and reproducible data collection, consistent with the PEO framework and the review objectives.

From each included source, the following data items were extracted.

- Bibliographic information: first author, year of publication, and DOI.
- Study characteristics: country of origin, study design (cross-sectional, Case–control, cohort, systematic review/meta-analysis, narrative review), and funding source.
- Population: sample size and participant characteristics (age range, sex distribution, clinical condition: chronic gastritis, gastric ulcer, gastric cancer, and asymptomatic carriers).
- Sample type: gastric biopsy (antrum/corpus/both), gastric fluid, stool sample, and others.
- Molecular diagnostic techniques: conventional PCR (target gene); qPCR (target gene, platform); 16S rRNA gene sequencing (hypervariable regions: V1–V2, V3–V4, V4, etc.; sequencing platform: Illumina MiSeq/HiSeq, Ion Torrent, 454 pyrosequencing); shotgun metagenomics (platform, sequencing depth); and others.
- Bioinformatics workflow: software used (QIIME, QIIME2, mothur, DADA2, MetaPhlAn, HUMAnN), reference databases (SILVA, Greengenes, NCBI), and taxonomic assignment methods (operational taxonomic units (OTUs), obtained through sequence clustering, versus amplicon sequence variants (ASVs), inferred through denoising methods).
- Main findings: alpha diversity metrics (Shannon, Simpson, Chao1, observed OTUs/ASVs), beta diversity metrics (UniFrac, Bray–Curtis), differential taxa at phylum and genus levels, assessed virulence factors (CagA, VacA, OipA, others), functional pathways (KEGG, MetaCyc), and identified antimicrobial resistance genes.
- Eradication data (if applicable): treatment regimen, duration of follow-up, and microbiota recovery outcomes.
- Reported limitations.

### 2.7. Assessment of Evidence Robustness

Consistent with PRISMA-ScR guidance, a formal risk-of-bias assessment (e.g., using tools such as the Newcastle–Ottawa Scale or QUADAS-2) was not conducted, as this scoping review was designed to map the breadth and characteristics of available evidence rather than to appraise intervention effects [26]. However, to provide readers with an informed basis for interpreting the strength of the reported findings, three proxy indicators of evidentiary robustness were systematically extracted and reported for each source: (a) study design (cross-sectional, Case–control, cohort, population-based, or synthesis/methodological), (b) sample size, and (c) evidence type classification (P: primary empirical; S: secondary synthesis; M: methodological), as detailed in Section 2.4.3. These indicators, summarized in Table 3 and discussed narratively in Section 3.2, allow the reader to weigh the relative strength of individual findings, particularly in instances where primary studies report contradictory results. Because total sample size was not consistently reported across all primary studies (Supplementary Table S1), sample-size descriptors were computed and interpreted only for studies with available numeric totals; therefore, these proxy indicators do not constitute a formal appraisal of methodological quality or risk of bias.

## 3. Results

### 3.1. Selection of Sources of Evidence

Sources for this review were selected using a systematic process involving database searches, duplicate removal, and bibliographic evaluation in accordance with PRISMA-ScR guidelines. In total, 180 records were identified across PubMed (*n* = 68), Scopus (*n* = 54), Web of Science (*n* = 37), and SciELO (*n* = 21). After removing 32 duplicates, 148 unique references underwent title and abstract screening. Of these, 84 were excluded for failing to meet at least one inclusion criterion. Sixty-four full-text articles were then assessed for eligibility, with 34 excluded due to insufficient methodological description (*n* = 12), lack of direct interaction between *H. pylori* and gastric microbiota (*n* = 9), inadequate description of molecular methods (*n* = 7), or inaccessibility of the full text (*n* = 6). Ultimately, 30 sources met all eligibility criteria and were included in the qualitative synthesis. Among these, 17 were primary empirical studies (cross-sectional, Case–control, cohort, and population designs) that generated original first-hand data, and 11 were secondary sources (*n* = 4 narrative reviews, *n* = 2 systematic reviews/meta-analyses, *n* = 3 international consensus documents, *n* = 2 critical reviews) that synthesize findings from multiple previous studies; and 2 were methodological studies included for their contribution to the technical characterization of low-biomass microbiota analysis, without providing specific data on *H. pylori* or the gastric microbiota (Figure 1).

### 3.2. Characteristics of Included Studies

The 30 included sources were published between 2006 and 2024, with the majority (*n* = 21) between 2016 and 2022. The sources are primarily from China (*n* = 10), Europe (*n* = 9; Austria, Germany, Italy, Portugal, Sweden), the Asia-Pacific region (*n* = 4; Japan, Malaysia), the United States (*n* = 3), and other regions (Mexico, United Arab Emirates; *n* = 4). Among the 17 primary studies, cross-sectional designs were the most common (*n* = 7), followed by longitudinal or eradication studies (*n* = 3), case–control studies (*n* = 3), population cohort studies (*n* = 2), interspecies comparative studies (*n* = 1), and basic descriptive studies (*n* = 1). Among primary empirical studies reporting total sample size (Supplementary Table S1), sample sizes ranged from 17 to 231 participants (median: 47). Gastric biopsies from the antrum, corpus, or both were the primary sample type in 12 studies. Fecal samples alone or in combination were used in 6 studies, and 4 studies used combined gastric and intestinal sampling.

In terms of evidentiary robustness, the 17 primary empirical studies varied substantially in design strength and sample size, factors that directly bear on the reliability of individual findings. Cross-sectional and Case–control designs (*n* = 10 combined) provide associative but not temporal evidence, whereas the three longitudinal/eradication studies [8,14,18] and the two population-based cohort studies [7,29] offer comparatively stronger designs for inferring temporal or exposure-related associations. Sample sizes varied widely across studies reporting total *n* (17 to 231 participants; median: 47), with several studies operating near the lower end of this range, which limits the statistical power and generalizability of their findings. This heterogeneity in design and sample size should be considered when interpreting apparently contradictory results, such as the divergent alpha-diversity trajectories reported after *H. pylori* eradication [8,14,18].

In the primary empirical studies, 16S rRNA gene sequencing was the most frequently employed molecular technique. One study used shotgun metagenomics, enabling taxonomic and functional analyses beyond amplicon resolution and incorporating both gastric and non-gastric (fecal) samples [7]. Another study combined quantitative PCR (qPCR) with 16S rRNA gene sequencing [8], enabling a complementary assessment of *Helicobacter pylori* targets and broader microbial community structures. Additionally, one study reported inferred functional analysis based on 16S rRNA data [27], which should be interpreted as functional potential rather than a direct metagenomic measure. To enable a transparent assessment of methodological heterogeneity, the primary empirical studies were examined separately from secondary and methodological sources. Table 3 provides a structured comparison of the 17 primary studies, including country, study design, sample type and anatomical site, molecular approach, sequencing target and platform, bioinformatic workflow/reference database, clinical condition, and reported sample size. This primary-study-focused approach avoids the inappropriate comparison of methodological variables that are not applicable to narrative reviews, meta-analyses, consensus documents, or methodological publications.

Substantial heterogeneity was evident across sampling strategies, molecular targets, sequencing platforms, and analytical pipelines. Most studies used 16S rRNA gene sequencing, but targeted different hypervariable regions and applied heterogeneous bioinformatic workflows and reference databases. In addition, the use of gastric biopsies, gastric fluid, duodenal aspirates, saliva, and stool samples reflects distinct anatomical compartments and prevents direct equivalence of microbiota profiles across studies. Total sample size was not consistently reported; therefore, sample-size descriptors in Table 3 are presented exactly as reported in the respective sources.

Of the 17 primary empirical studies, 13 reported alpha-diversity results. Of these, 11 showed significantly reduced alpha diversity (Shannon and/or Chao1 indices) in *Helicobacter pylori*-positive individuals compared to *Helicobacter pylori*-negative controls. One study reported increased diversity after eradication treatment [8], and another observed high inter-individual variability without a consistent directional change [14]. This apparent contradiction should be interpreted in light of differences in study design and sample size (Section 2.7): ref. [8] was a longitudinal study with prospective follow-up (*n* = 47), whereas [14] employed a smaller cross-sectional sample, a distinction that may partly account for the divergent directionality observed.

At the phylum level, a higher relative abundance of *Proteobacteria* in *Helicobacter pylori*-positive gastric samples was the most consistent finding [3,5,9], accompanied by decreases in the abundances of *Firmicutes* and *Actinobacteria* [4,12]. At the genus level, *Fusobacterium*, *Peptostreptococcus*, and *Clostridium* were repeatedly enriched in advanced gastric lesions [5,16,21], while the relative abundance of *Helicobacter pylori* decreased in gastric carcinoma tissues compared to early lesion stages [16]. Of the 30 included sources, 15 evaluated or mentioned virulence factors, primarily *CagA*, *VacA*, and *OipA*. Only one study [11] used metatranscriptomics to assess active transcriptional expression, providing direct in vivo evidence of virulence gene activity during gastric infection.

### 3.3. Advances Enabled by Molecular Diagnostic Approaches

Molecular diagnostic techniques have been fundamental in advancing the understanding of *H. pylori* and the gastric microbiota. Traditional polymerase chain reaction (PCR) allows for rapid and economical detection of *H. pylori* by amplifying specific target genes (*ureA*, *glmM*, 16S rRNA), offering high diagnostic sensitivity and low operating costs [4,20]. However, this approach does not allow for bacterial quantification or functional characterization.

Quantitative real-time PCR (qPCR) extends the detection offered by traditional PCR by enabling precise quantification of bacterial load, identification of virulence genes (*cagA*, *vacA*, *oipA*, *babA2*), and detection of point mutations associated with clarithromycin resistance in the 23S rRNA gene (*A2142G*, *A2143G*) and fluoroquinolone resistance in *gyrA* [8,20,27]. The application of qPCR to gastric biopsies and fecal samples increases its clinical utility. One of the reviewed studies combined qPCR with 16S rRNA gene sequencing, demonstrating the complementary strengths of these methods for simultaneous assessment of pathogenic load and microbial community structure [8], while another used a quantitative DNA/RNA-based approach in parallel with 16S profiling to assess both viable bacterial communities and specific targets [23].

In this regard, 16S rRNA gene sequencing has facilitated the identification of non-culturable microorganisms and the characterization of taxonomic variations related to *H. pylori* infection and the progression of gastric diseases. Among the 13 primary studies that used 16S rRNA gene sequencing, the targeted hypervariable regions varied substantially. The V3–V4 region was most frequently used (*n* = 9), followed by V1–V2 (*n* = 4), V4 (*n* = 2), and V3 (*n* = 1); some studies assessed more than one region.

Shotgun metagenomic sequencing offers comprehensive coverage by enabling simultaneous taxonomic and functional profiling, including reconstruction of metabolic pathways and identification of antimicrobial resistance genes, without the taxonomic resolution limitations of amplicon-based methods [7]. However, its bioinformatics complexity and high cost limit its use in the primary studies reviewed. One study employed functional profiling tools based on 16S rRNA data as a more cost-effective alternative [27]. The accuracy of these tools depends on the completeness of reference databases and may not fully leverage the functional diversity of low-biomass upper gastrointestinal tract communities [24]. The main applications, advantages, and limitations of each molecular approach are summarized in Table 4.

### 3.4. Diagnostic Performance and Clinical Applicability of Molecular Methods

From a diagnostic performance standpoint, conventional PCR offers high analytical sensitivity for the detection of *Helicobacter pylori* through amplification of specific target genes (*ureA*, *glmM*, *16S rRNA*) at low operational cost, but does not permit bacterial quantification, resistance profiling, or functional characterization, limiting its clinical utility to qualitative detection [4,20]. Quantitative real-time PCR (qPCR) extends this capability by enabling precise quantification of bacterial load and, critically, the direct detection of point mutations associated with clarithromycin resistance (*23S rRNA*, *A2142G/A2143G*) and fluoroquinolone resistance (*gyrA*) [8,20,27], properties that directly inform empirical versus susceptibility-guided treatment decisions [20]. Despite this recognized capability, none of the primary studies included in this review directly evaluated the diagnostic accuracy (sensitivity, specificity) of qPCR-based resistance detection against phenotypic susceptibility testing as a reference standard, representing a substantial gap for clinical implementation.

16S rRNA gene sequencing provides broader taxonomic characterization and the detection of unculturable microorganisms, expanding diagnostic scope beyond single-pathogen detection; however, its clinical applicability is constrained by limited species-level resolution and dependence on inferential tools (e.g., PICRUSt2) for functional prediction, which estimate rather than directly measure metabolic or resistance-related activity [24,27]. Shotgun metagenomics offers the highest taxonomic and functional resolution, including direct identification of antimicrobial resistance genes without amplification bias [7]; nonetheless, its bioinformatic complexity, cost, and susceptibility to contamination in low-biomass gastric samples currently restrict its integration into routine clinical workflows [24].

Regarding standardization, the absence of harmonized protocols for DNA extraction, choice of 16S hypervariable region, sequencing platform, and bioinformatics pipeline (OTU-based clustering versus ASV denoising) constitutes a major barrier to the clinical implementation of these molecular tools, as it precludes the establishment of standardized diagnostic thresholds or reference values applicable across laboratories [11,23,24]. Taken together, these findings indicate that while molecular diagnostic tools have expanded pathogen detection and resistance profiling capabilities, rigorous head-to-head diagnostic accuracy studies and standardized implementation protocols remain notably absent from the current evidence base.

### 3.5. Eradication of H. pylori and Effects on Microbiota

Multiple sources have examined the microbiological consequences of *Helicobacter pylori* eradication therapy [3,8,12,16]. Both triple therapy, which typically includes a proton pump inhibitor, clarithromycin, and amoxicillin, and quadruple regimens initially reduce bacterial diversity due to the broad-spectrum effects of antibiotics on commensal microorganisms [3,8]. A longitudinal study [8] found that eradication in asymptomatic young adults was associated with recovery, rather than disruption, of the gastrointestinal microbiota, with partial restoration of alpha diversity observed within four weeks after treatment. In contrast, a stool-based high-throughput 16S rRNA gene amplicon sequencing study [18] identified significant alterations in the gut microbiota following eradication, including increased Proteobacteria and decreased Bifidobacterium in stool samples. These findings suggest that responses across compartments may differ substantially. A recent critical review [3] reported that microbiota recovery after eradication is often transient and incomplete, with some patients experiencing persistent changes that may reflect pre-existing dysbiosis or prolonged antibiotic effects. The use of probiotics, particularly *Lactobacillus* and *Bifidobacterium* strains, has been proposed as an adjunctive strategy to promote microbial recovery and reduce treatment-associated adverse effects [3]. However, none of the primary studies included in this review specifically evaluated probiotic interventions, highlighting the need for dedicated prospective research. Key microbiological findings related to *H. pylori* from the included sources are summarized in Table 5.

## 4. Discussion

Beyond confirming dysbiosis patterns already described in the literature, this review provides an explicit comparative synthesis of the performance, applicability, and limitations of the molecular diagnostic tools used in the included studies (Table 4), as well as the identification of a previously unreported methodological gap: the absence of primary studies that directly compare the diagnostic performance (sensitivity, specificity) of qPCR against a reference standard in the same population.

The findings synthesized in this review should primarily be interpreted as associations rather than as evidence of direct causality. Most primary empirical studies were cross-sectional or case–control investigations and therefore cannot establish temporality, rule out reverse causation, or fully control for confounding factors, including proton pump inhibitor use, prior antibiotic exposure, dietary patterns, age, smoking, alcohol consumption, other host characteristics, clinical stage, and anatomical sampling site. Although longitudinal and eradication studies provide stronger evidence of temporal associations, they do not, by themselves, establish that *H. pylori* is the sole determinant of the observed differences in the microbiota. Accordingly, the observed patterns should be interpreted as *H. pylori* status-associated microbial signatures within heterogeneous clinical and methodological contexts.

*Helicobacter pylori* colonization is typically associated with alterations in the gastric microbial ecosystem and dysbiotic patterns reported at various stages of the Correa cascade of disease. The decrease in alpha diversity and the predominance of *Proteobacteria* in *H. pylori*-positive individuals, observed in geographically diverse primary studies using 16S rRNA sequencing and shotgun metagenomics [7,9,10,15,16,28,31], are among the most commonly reported patterns in the available evidence. These findings are consistent with proposed pathophysiological models in which *H. pylori*-associated changes in the acidic microenvironment, epithelial inflammation, and immunomodulation may create ecological conditions compatible with the observed microbial patterns, thereby favoring microbial taxa adapted to inflammatory conditions [28].

Virulence factors represent a crucial mechanistic link between *H. pylori* infection and alterations in the microbiome. CagA has been implicated in alterations in host–cell signaling that may contribute to changes in the gastric microenvironment; however, the observational microbiome studies included do not establish that these mechanisms directly cause dysbiosis [13,14]. VacA has experimentally documented cellular effects, including vacuolization, mitochondrial dysfunction, and immunomodulation; their direct contribution to the microbiota patterns reported in human observational studies remains to be established [18,19]. Notably, the only study in this review that employed metatranscriptomics [11] reported active in vivo expression of *cagA*, *vacA*, *babA2*, and *oipA* during infection, providing direct evidence of virulence gene activity rather than just their presence, a distinction of considerable clinical importance that should be replicated in larger cohorts.

Although qPCR is widely recognized for its ability to detect resistance-conferring mutations in the 23S rRNA and *gyrA* genes [20,27], none of the primary studies in this mapping directly compared the diagnostic performance (sensitivity, specificity) of qPCR with a reference standard in the same population. Similarly, while shotgun metagenomics offers unparalleled functional resolution [7], its application to gastric samples is limited by cost and the inherent contamination challenges of low-biomass environments [24]. The combination of qPCR and 16S rRNA sequencing, as employed in [8,23], represents a pragmatic middle ground that should be more widely applied in clinical trials.

Methodological heterogeneity proved to be the greatest limitation for comparability across studies. In the 13 primary studies using 16S sequencing, at least four different hypervariable regions were targeted, three distinct reference databases were employed (SILVA, Greengenes, RDP), and bioinformatics pipelines varied from OTU-based clustering (QIIME) to ASV denoising (QIIME2/DADA2), methods that are not directly comparable in their taxonomic resolution [11,23,24]. Furthermore, the concomitant use of proton pump inhibitors is a major confounding factor for gastric microbiome composition, regardless of *H. pylori* status [17], but was not systematically controlled in most of the included primary studies, limiting the interpretability of community differences attributed solely to infection.

Gastric preneoplastic lesions, particularly chronic atrophic gastritis and intestinal metaplasia, may also contribute substantially to the heterogeneity observed across studies of the gastric microbiome. These conditions have been associated with changes in gastric microbial diversity and composition, potentially related to alterations in gastric acidity, mucosal architecture, and the local inflammatory environment. However, it remains challenging to determine whether these microbiome changes are directly driven by *H. pylori* infection, by the preneoplastic lesions themselves, or by their interaction. Therefore, future studies should systematically characterize and stratify participants by histological stage, including the presence and severity of atrophy and intestinal metaplasia, to improve comparability and clarify the independent contributions of these features to gastric microbial profiles.

Another source of heterogeneity concerns sample types and anatomical sites. Studies using only the antrum, only the corpus, or both in gastric biopsies yielded different results, reflecting actual biogeographical variability within the stomach, and these differences should be considered in study design [3,9,30]. Several included studies exclusively used stool samples or in combination with gastric tissue [7,8,18,19,29]. Although these provide valuable information on the systemic effects of the microbiome, they cannot be directly equated with data from the gastric mucosal community and were treated separately in this comprehensive analysis.

Regarding the efficacy of eradication therapy, available evidence suggests a generally restorative effect on the gastric microbiome [8], although the magnitude and duration of recovery vary depending on the antibiotic regimen, follow-up period, and initial microbiome composition [3,12]. The potential adjuvant role of probiotics in supporting microbiome recovery is biologically plausible and clinically promising, but it was not evaluated in any primary study within this mapping, representing a clear gap for future research.

### 4.1. Future Directions and Emerging Technologies

Future research should move beyond predominantly cross-sectional, 16S rRNA gene-based descriptions toward prospective and standardized designs capable of evaluating temporal microbiome dynamics. Repeated sampling before, during, and after *H. pylori* eradication, together with systematic documentation of proton pump inhibitor exposure, antibiotic use, diet, clinical phenotype, and exact biopsy location (antrum, corpus, or both), would improve the assessment of temporal associations and reduce residual confounding.

Metatranscriptomics represents a particularly relevant approach because it can distinguish transcriptionally active microbial and virulence-related signals from genomic potential alone. In the present mapping, only one primary study applied this approach [11], underscoring the need for replication in larger and clinically diverse cohorts. Similarly, functional predictions derived from 16S rRNA gene data should be interpreted with caution because they reflect functional potential rather than directly measuring gene expression or metabolite production [27].

Integrated multi-omics frameworks combining shotgun metagenomics, metatranscriptomics, metabolomics, and host-derived data may provide a more comprehensive characterization of host–microbe interactions in the gastric niche. Strain-level analysis, particularly when supported by high-resolution shotgun sequencing, may further clarify whether specific *H. pylori* lineages, virulence profiles, antimicrobial resistance determinants, or non-Helicobacter strains are associated with distinct ecological signatures or clinical phenotypes. Because gastric samples are low-biomass specimens, these approaches should incorporate rigorous negative controls, contamination-aware analytical workflows, and transparent reporting of laboratory and bioinformatic procedures [24].

Collectively, future studies should prioritize multicenter longitudinal cohorts, harmonized sampling and sequencing protocols, paired gastric and non-gastric specimens when clinically justified, and pre-specified analytical pipelines. Such studies are required to distinguish reproducible associations from potential causal pathways and to evaluate the translational relevance of molecular microbiome profiling for diagnosis, risk stratification, and treatment monitoring.

### 4.2. Limitations

Among the methodological limitations, the following should be considered: first, the bibliographic search was limited to four electronic databases (PubMed, Scopus, Web of Science, and SciELO) and to publications in English and Spanish; studies published in other languages or indexed exclusively in regional databases may not have been included. Second, a formal critical evaluation of methodological quality or risk of bias (e.g., using standardized tools such as the Newcastle–Ottawa Scale) was not performed, consistent with the exploratory objective of scoping reviews [26], which prioritize the breadth of evidence mapping over the assessment of intervention effectiveness. To partially mitigate this limitation, study design, sample size, and evidence type (P/S/M) were systematically charted, with the methodological characteristics of the 17 primary empirical studies summarized in Table 3 (Section 2.7) and used as proxy indicators to contextualize the relative strength of individual findings; however, this approach does not substitute for a formal risk-of-bias appraisal and should be considered when interpreting the reported associations. Third, publication bias must be considered, as studies with positive or statistically significant results are more likely to be published than those with null results. Finally, the methodological heterogeneity observed among the included studies, in DNA extraction protocols, 16S hypervariable regions, bioinformatics pipelines, and reference databases, limited the direct comparability of the reported results and prevents a quantitative synthesis.

An additional limitation relates to the diversity of evidence types included in the qualitative synthesis. The included sources encompass primary empirical studies, narrative and systematic reviews, international consensus documents, and methodological studies. While chosen for their inclusive and exploratory nature [26], scoping reviews introduce inherent differences in how the synthesized evidence is interpreted. Narrative reviews and consensus documents [2,4,25] provide information synthesized and interpreted by experts rather than original data, which can introduce selection and interpretation biases that are difficult to quantify. Similarly, the two included methodological studies [6,24] do not provide direct evidence on *H. pylori*–gastric microbiome interactions; their role in this review was limited to contextualizing the technical landscape of molecular approaches applied to low-biomass gastric samples. Finally, the predominance of studies conducted in Asian populations, particularly in China and Japan, may limit the generalizability of the reported results across regions, as host genetic factors, dietary habits, and *H. pylori* strain diversity vary considerably.

## 5. Conclusions

Across the included literature, *Helicobacter pylori* infection status was consistently associated with differences in the gastric microbial ecosystem; however, the available evidence does not establish direct causation. *H. pylori* colonization was frequently associated with lower alpha diversity, a higher relative abundance of *Proteobacteria*, including *Helicobacter*, and lower relative abundances of *Firmicutes*, *Actinobacteria*, and *Bacteroidetes*. Nevertheless, the magnitude and direction of these patterns varied across studies and may have been influenced by methodological heterogeneity, clinical characteristics, anatomical sampling site, proton pump inhibitor use, previous antibiotic exposure, and other potential confounders.

Virulence factors, particularly CagA and VacA, are biologically plausible contributors to host–microbe interactions in the gastric environment. Their experimentally observed effects on epithelial signaling, cellular injury, and immune modulation may be relevant to the ecological conditions associated with the microbial patterns reported in human studies; however, the available observational evidence does not establish a direct causal role for them in gastric dysbiosis or disease progression. Active transcriptional expression of cagA, vacA, babA2, and oipA was reported in one metatranscriptomic study [11], supporting in vivo virulence gene activity during infection; this observation requires replication in larger and more diverse cohorts. Molecular diagnostic tools have significantly expanded our ability to investigate *H. pylori* and the gastric microbiome. This comparative synthesis of molecular diagnostic tools, together with the explicit classification of the type of evidence used, constitutes a complementary methodological contribution to previous reviews focused on the biological mechanisms of gastric dysbiosis. PCR and qPCR offer high sensitivity for pathogen detection, virulence gene identification, and antibiotic resistance analysis; 16S rRNA sequencing allows for community-level taxonomic characterization; and shotgun metagenomics provides comprehensive functional resolution. The complementary use of these methods is encouraged to gain both pathogen-specific and community-wide insights in a single study.

However, significant methodological heterogeneity exists in hypervariable region selection, bioinformatics pipelines, reference databases, and contamination, which continues to limit inter-study comparability and the reproducibility of reported findings. Standardization of molecular protocols, systematic reporting of potential confounders (proton pump inhibitor use, antibiotic exposure, anatomical sampling sites), and inclusion of extraction negative controls are crucial steps toward building a more coherent evidence base in this field.

Finally, combining advanced molecular techniques with well-designed longitudinal clinical studies is essential to elucidate the causal mechanisms between *H. pylori*, microbiome dysbiosis, and the progression of gastric diseases, and to develop more precise diagnostic and treatment strategies.

## Figures and Tables

**Figure 1:**
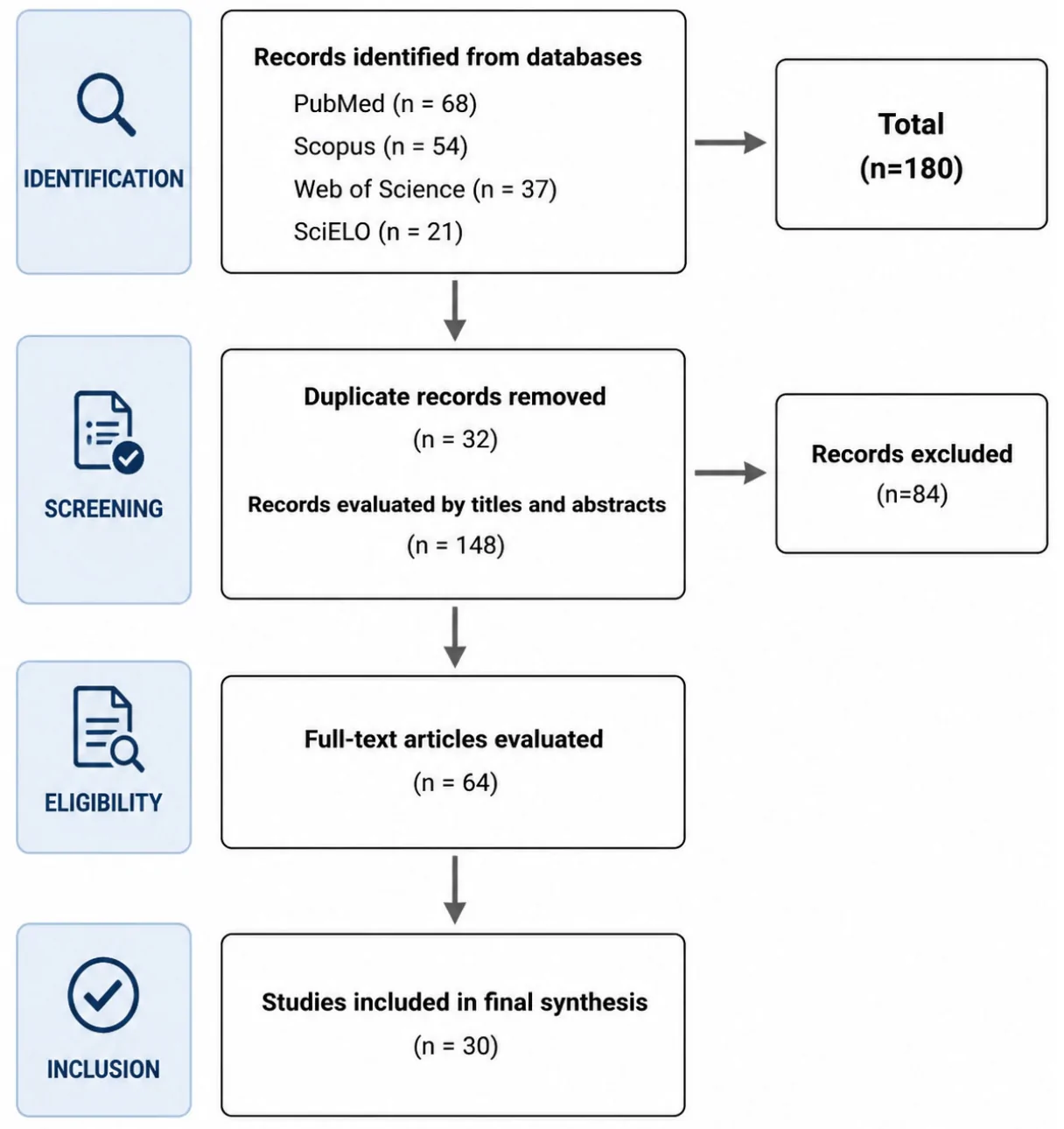
PRISMA-ScR flow diagram of the study selection process. A total of 180 records were identified through electronic database searches (PubMed, *n* = 68; Scopus, *n* = 54; Web of Science, *n* = 37; SciELO, *n* = 21). After removing 32 duplicates, 148 records were screened by title and abstract, and 84 were excluded at this stage. Sixty-four full-text articles were assessed for eligibility; thirty-four were excluded with documented reasons. Finally, 30 sources were included in the qualitative synthesis. Flowchart generated using the PRISMA flow diagram tool (https://estech.shinyapps.io/prisma_flowdiagram/, accessed on 13 July 2026).

**Table 1:** Bibliographic Search Strategy and Results Obtained.

Database	Search Strategy	Field	Filters	Results
PubMed	(“*Helicobacter pylori*”[MeSH] OR “*Helicobacter pylori*”[tiab]) AND (“gastrointestinal microbiome”[MeSH] OR “gastric microbiota”[tiab] OR “gastric microbiome”[tiab] OR “gastric dysbiosis”[tiab]) AND (“molecular diagnostic techniques”[MeSH] OR “PCR”[tiab] OR “qPCR”[tiab] OR “16S rRNA”[tiab] OR “metagenomics”[tiab] OR “next-generation sequencing”)	All fields	English/Spanish; 2006–2025	68
Scopus	TITLE-ABS-KEY (“*Helicobacter pylori*” AND (“gastric microbiome” OR “gastric microbiota” OR “gastric dysbiosis”) AND (“16S rRNA” OR “metagenomics” OR “qPCR” OR “virulence factors”))	Title/Abtract/Keywords	2006–2025; Article/Review	54
Web of Science	TS = (“*Helicobacter pylori*” AND (“shotgun metagenomics” OR “16S rRNA sequencing” OR “qPCR”) AND (“gastric microbiota” OR “gastric microbiome”))	Topic	2006–2025	37
SciELO	(“*Helicobacter pylori*” AND “microbiota gástrica”) OR (“diagnóstico molecular” AND “disbiosis gástrica”)	All fields	2006–2025	21
Total	—			180

**Table 2:** Examples of Key Studies Included in the Review.

Author/Year	Country	Study Design	Molecular Technique	Sample	Principal Finding	Evidence Type
Guo et al., 2020 [18]	China	Population-based cohort	Shotgun metagenomics (WGS)	Gastric biopsy + stool (*n* = 105)	*H. pylori*-associated dysbiosis and *Proteobacteria* predominance linked to premalignant gastric lesions	P
He et al., 2019 [6]	China	Longitudinal prospective	qPCR + 16S rRNA gene sequencing	Gastric biopsy + stool (*n* = 47)	Partial restoration of gastrointestinal microbiota following *H. pylori* eradication	P
Klymiuk et al., 2017 [7]	Austria	Cross-sectional observational	16S rRNA gene sequencing (V1–V2)	Gastric biopsy (Antrum + Corpus) (*n* = 30)	*Proteobacteria* predominance; reduced diversity in *H. pylori*-positive individuals	P
Liu et al., 2022 [11]	China	Meta-analysis (secondary 16S data)	NGS/16S rRNA	Biopsies from 12 primary studies	Reduced Firmicutes; universal dysbiotic signatures along the Correa cascade	S
Coker et al., 2018 [23]	China	Case–control	16S rRNA Sequencing (V3–V4)	Gastric mucosal biopsy, Antrum (*n* = 81)	Progressive dysbiosis related to gastric carcinogenesis; *Fusobacterium* and *Peptostreptococcus*	P

Note: Selected representative sources are shown. The complete evidence map of all included sources is provided in Supplementary Table S1. P, primary empirical study; S, secondary source.

**Table 3:** Structured Methodological Comparison of Primary Empirical Studies Evaluating *Helicobacter pylori* and the Gastric Microbiota (*n* = 17).

Study (Author, Year)	Ref.	Country	Study Design	Sample Type/ Anatomical Site(s)	Main Molecular Technique	Target Gene/16S Region	Sequencing Platform	Bioinformatic Pipeline/ Reference Database	Clinical Condition	Total *n* (as Reported)
Guo et al., 2020	[18]	China	Cohort/ population-based	Gastric biopsy + stool	Shotgun metagenomics	Microbial whole genome	Illumina HiSeq	QIIME2/MetaPhlAn2/HUMAnN2/NCBI	Premalignant lesions in high-risk populations	231 (Linqu subcohort)
He et al., 2019	[6]	China	Prospective longitudinal	Gastric biopsy + stool	qPCR + 16S rRNA	V3–V4	Illumina MiSeq	QIIME/Greengenes	Asymptomatic carriers	47
Klymiuk et al., 2017	[7]	Austria	Multicenter Cross-sectional	Biopsy (Antrum + Corpus) + saliva + duodenal aspirate	16S rRNA	V1–V2	Illumina MiSeq	QIIME/SILVA 119	Dyspepsia/gastritis/controls	30 (11 *H. pylori*+; 19 *H. pylori*−)
Coker et al., 2018	[23]	China (Hong Kong)	Case–control	Mucosal biopsy (antrum)	16S rRNA	V3–V4	Illumina MiSeq	QIIME/SILVA/LEfSe	Correa’s cascade	81 (multiple stages)
Sterbini et al., 2016	[24]	Italy	Cross-sectional	Mucosal biopsy (antrum)	16S rRNA	V3–V4	Illumina MiSeq	QIIME/Greengenes 13_8	Dyspepsia; proton pump inhibitor use	Not reported
Brawner et al., 2017	[12]	USA	Pediatric Cross-sectional	Gastric biopsy (antrum)	16S rRNA	V4	Illumina MiSeq	QIIME/Greengenes/LEfSe	Pediatric infection	44 (22 + 22)
Schulz et al., 2018	[27]	Germany	Cross-sectional (RNA-based)	Upper GI biopsies (multiple sites)	16S rRNA (RNA-based)	V1–V2	Illumina MiSeq	QIIME/SILVA/UniFrac	Upper GI tract; includes H. heilmannii	35 (18 with *Helicobacter)*
Yap et al., 2016	[25]	Malaysia	Longitudinal	Stool	high-throughput 16S rRNA gene sequencing	V3–V4	Illumina MiSeq	QIIME pipeline.	*H. pylori*-positive young adults undergoing eradication therapy	**26**
Dash et al., 2019	[20]	United Arab Emirates	Cross-sectional	Stool + gastric biopsy	16S rRNA	V3–V4	Illumina MiSeq	QIIME2/SILVA/LEfSe	Adults *H. pylori+*	52 (26 + 26)
Ferreira et al., 2018	[17]	Portugal	Case–control	Biopsy (antrum + corpus)	16S rRNA	V3–V4	Illumina MiSeq	QIIME/SILVA/LEfSe	Gastric cancer vs. gastritis	54 (27 + 27)
Gao et al., 2018	[21]	China	Cross-sectional	Stool	16S (stool)	V4	Illumina MiSeq	QIIME/Greengenes	Premalignant lesions (association)	47
Chen et al., 2019	[28]	China	Case–control (paired)	Tumor and adjacent tissue (pairs)	16S rRNA	V3–V4	Illumina MiSeq	QIIME2/SILVA/LEfSe	Gastric cancer	62 pairs
Jiang et al., 2021	[22]	Japan	Observational	Gastric mucosa + gastric fluid	16S rRNA	V3–V4	Illumina MiSeq	QIIME2/SILVA	Chronic antral gastritis	Not reported
Maeda et al., 2022	[26]	Japan	Observational	Gastric biopsy + duodenal aspirate	16S + functional prediction	V1–V2	Illumina MiSeq	QIIME2/PICRUSt2/KEGG	Chronic gastritis	Not reported
Bik et al., 2006	[5]	USA	Foundational descriptive study	Biopsy (antrum/corpus)	16S (454)	V1–V3 and V6–V9	454 pyrosequencing	RDP classifier	Healthy stomach	23 (9 *H. pylori*+; 14 *H. pylori*−)
Thorell et al., 2017	[9]	Sweden	In vivo (metatranscriptomics + 16S)	Mucosal biopsy (corpus)	RNA-seq + 16S	V3 + total RNA	Illumina HiSeq 2500	QIIME/Bowtie2/DESeq2/SILVA	Active infection	Not reported
Wurm et al., 2018	[15]	Austria	Comparative multispecies study	Human biopsy + animal models	qPCR + 16S (DNA/RNA)	V1–V2	Illumina MiSeq	QIIME/SILVA 128	*H. pylori* infection (human/animal)	Not reported

Note: Only primary empirical studies generating original data were included. qPCR, quantitative polymerase chain reaction; GI, gastrointestinal; KEGG, Kyoto Encyclopedia of Genes and Genomes; LEfSe, linear discriminant analysis effect size. “Not reported” indicates that the total sample size was not reported in the source from which the data were extracted (Supplementary Table S1). Sample-size descriptors are presented as reported by the original studies and may refer to participants, study subcohorts, or paired specimens.

**Table 4:** Comparison of Molecular Techniques Used in Studies on *Helicobacter pylori* and Gastric Microbiota.

Molecular Technique	Principal Applications	Advantages	Limitations	Diagnostic Performance Traits	Clinical Implementation Considerations
Conventional PCR	Detection of *H. pylori* and bacterial genes	High diagnostic sensitivity and low operational cost	Does not permit bacterial quantification or functional analysis	High analytical sensitivity for qualitative detection; not reported quantitatively in the included studies	Low cost, requires minimal infrastructure; does not allow resistance profiling.
qPCR	Bacterial quantification; detection of virulence genes (*cagA*, *vacA*, *oipA*) and antimicrobial resistance mutations (23S rRNA, *gyrA*)	High sensitivity, real-time specificity, and applicability to clinical samples (biopsy and stool)	Requires specialized equipment and higher technological cost; limited to pre-defined targets	Direct detection of resistance mutations (23S rRNA, gyrA); sensitivity/specificity not evaluated against the reference standard in the included studies.	Applicable to biopsy and stool samples; requires specialized equipment; no head-to-head diagnostic validation studies.
16S rRNA Sequencing	Taxonomic characterization of gastric microbiota; identification of non-cultivable microorganisms	Captures community-level diversity including unculturable taxa; widely applicable	Limited species-level resolution; functional profiling requires inference tools that estimate, but do not directly measure, functional capacity	Broad taxonomic resolution; inferred (not directly measured) functionality.	Requires standardization of the hypervariable region and bioinformatics pipeline before clinical implementation.
Shotgun Metagenomics	Comprehensive taxonomic and functional microbiome analysis; detection of metabolic pathways, virulence and antimicrobial resistance genes	Highest taxonomic resolution; direct functional profiling without amplification bias	High bioinformatic complexity and cost; in low-biomass gastric samples, contamination risk is critical if negative extraction controls are not systematically applied [24]	Higher functional and resistance resolution; no amplification bias.	High cost, bioinformatics complexity, and the risk of contamination in low-biomass samples limit its routine use.

**Table 5:** Principal Microbiological Findings Associated with *Helicobacter pylori*.

Author/ Year	Study Type/ Molecular Technique	Sample	Principal Microbiological Findings	Evidence Type
Guo et al., 2020 [18]	Shotgun metagenomics	Gastric biopsy + Stool (*n* = 105)	Proteobacteria predominance and alterations related to premalignant gastric lesions	P
Serrano et al., 2021 [2]	Narrative review (PCR/qPCR/NGS)	Multiple clinical studies	Overview of *H. pylori*-induced alterations in gastric microbial balance and host–microbiota interactions	S
Jiang et al., 2021 [22]	16S rRNA gene sequencing (V3–V4)	Gastric mucosa + gastric fluid (chronic antral gastritis)	Differential bacterial community structure between mucosal and luminal gastric compartments	P
Ji et al., 2021 [30]	Narrative review	Gastrointestinal studies	Relationship between gastric dysbiosis and gastrointestinal diseases	S
Liu et al., 2022 [11]	Systematic review/Meta-analysis (16S NGS)	Biopsies from 12 primary studies	Decreased bacterial diversity; universal dysbiotic signatures along the Correa cascade	S
Maeda et al., 2022 [26]	16S rRNA gene sequencing (V1–V2) + inferred functional profiling (PICRUSt2/KEGG)	Gastric biopsy + duodenal aspirate	Metabolic pathway alterations and taxonomic shifts associated with *H. pylori* infection	P
Garrido Treviño et al., 2022 [19]	Narrative review/Molecular diagnosis	Therapeutic studies	Utility of molecular tools for detection and antimicrobial resistance profiling	S
Li et al., 2024 [3]	Narrative/ Critical review	Recent molecular studies	Transient and incomplete microbiota recovery following *H. pylori* eradication	S
Malfertheiner et al., 2023 [4]	International clinical consensus	Multicentric evidence	Clinical management of *H. pylori*; relationship between chronic infection and gastric disease progression	S

## Data Availability

No new data were created or analyzed in this study. Data sharing is not applicable to this article.

## Supplementary Materials

The following supporting information can be downloaded at: https://www.mdpi.com/article/10.3390/microorganisms14102137/s1, Table S1: Previous Studies Data.

## Abbreviations

ASVs	Amplicon Sequence Variants
CagA	Cytotoxin-associated gene A
DNA	Deoxyribonucleic Acid
GI	Gastrointestinal
Hp	*Helicobacter pylori*
IL	Interleukin
NCBI	National Center for Biotechnology Information
NGS	Next-Generation Sequencing
OipA	Outer Inflammatory Protein A
WHO	World Health Organization
OR	Odds Ratio
OTUs	Operational Taxonomic Units
PCR	Polymerase Chain Reaction
qPCR	Quantitative Polymerase Chain Reaction
RNA	Ribonucleic Acid
ROS	Reactive Oxygen Species
rRNA	Ribosomal RNA
UBT	Urea Breath Test
VacA	Vacuolating Cytotoxin A
16S rRNA	16S Ribosomal RNA
